# Doppler Navigation System with a Non-Stabilized Antenna as a Sea-Surface Wind Sensor

**DOI:** 10.3390/s17061340

**Published:** 2017-06-09

**Authors:** Alexey Nekrasov, Alena Khachaturian, Vladimir Veremyev, Mikhail Bogachev

**Affiliations:** 1 Department of Radio Engineering Systems, Saint Petersburg Electrotechnical University, Professora Popova 5, Saint Petersburg 197376, Russia; khachaturyan.al@gmail.com (A.K.); ver_vi@mail.ru (V.V.); rogex@yandex.com (M.B.); 2 Institute for Computer Technologies and Information Security, Southern Federal University, Chekhova 2, Taganrog 347922, Russia; 3 Faculty of Electrical Engineering and Informatics, Technical University of Košice, Letná 9, Košice 04200, Slovakia

**Keywords:** Doppler navigation system, normalized radar cross section, scatterometer, sea wind, algorithm

## Abstract

We propose a concept of the utilization of an aircraft Doppler Navigation System (DNS) as a sea-surface wind sensor complementary to its normal functionality. The DNS with an antenna, which is non-stabilized physically to the local horizontal with *x*-configured beams, is considered. We consider the wind measurements by the DNS configured in the multi-beam scatterometer mode for a rectilinear flight scenario. The system feasibility and the efficiency of the proposed wind algorithm retrieval are supported by computer simulations. Finally, the associated limitations of the proposed approach are considered.

## 1. Introduction

Microwave backscatter from the sea/ocean surface and remote measurement of the sea-surface wind have been actively investigated during the last seven decades [1,2,3,4,5,6,7,8,9,10,11,12,13,14,15,16,17,18,19,20,21]. The sea clutter is usually described by the normalized radar cross section (NRCS) and its statistical characteristics including probability distributions, spectral shapes of the Doppler reflections and the amplitude correlations [22]. For studying sea clutter and recovering the sea-surface wind vector, radars operating as scatterometers are rather common tool. Fundamentals of the scatterometer wind retrieval are based on the geophysical model function generated from the backscatter measurements collected from airborne and/or spaceborne platforms over the observed sea/ocean areas where wind fields are available simultaneously from various independent sources. The accuracy of scatterometer measurements of the wind direction is about ±20°, while the accuracy of the wind speed estimation is about ±2 m/s for wind speeds between 3 and 24 m/s, respectively.

Such measurements with airborne scatterometers are usually performed by either a measuring instrument with a fixed fan-beam antenna at the circular track flight or a radar with a rotating antenna at the rectilinear track flight. However, the size of a microwave antenna with a narrow beam is considerable at Ku-, X- and C-bands. Though recent advancement in antenna technologies allow to make them small enough to be installed on aircraft as a separate piece of equipment [23], installation of any additional equipment, especially on small aircraft, has its own drawbacks such as the reduction of aerodynamic characteristics, need for additional power supply etc. Accordingly, the most straightforward solution would be to also use the navigation equipment already available onboard for sea surface monitoring, in addition to its normal functionality.

One promising airborne radar instrument for such an application is the DNS. The system is equipped either with a fixed antenna, which is the most common solution in modern DNS designs, or with a track-stabilized antenna with additional its roll and pitch stabilization [24]. The DNS operation with a fixed-antenna system appears more difficult due to lack of its track stabilization. Similar situation can also take place in the case of track-stabilized antenna system when current roll or pitch angles exceed maximum angles of stabilization. The above setting is the particular focus of this article.

In this paper, we suggest the complementary utilization of the onboard DNS equipped with a fixed antenna system (non-stabilized relative to the local horizontal) as a sea-surface wind sensor in addition to its normal functionality. We also propose a particular algorithm for the sea surface wind speed and direction retrieval from the DNS measurements. This new application of the DNS as a sensor of the sea-surface wind, in addition to its typical navigational functions, is achieved in a scatterometer mode when the system operates as a four-beam scatterometer.

## 2. Materials and Methods

### 2.1. Doppler Navigation System Overview

**Figure 1:**
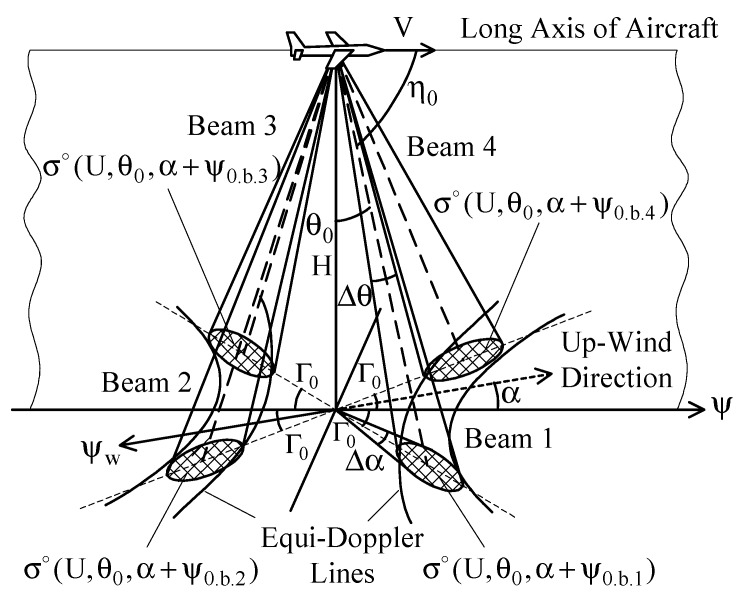
The typical beams geometry of the DNS: *λ*-configured beams 1, 2, and 3; *x*-configured beams 1, 2, 3, and 4 [27].

DNS is a completely self-contained radar system for measuring the aircraft ground speed and drift based on the Doppler principle and providing the dead-reckoning navigation of aircraft [25]. Additional navigation input originating from DNS helps to eliminate several problems associated with early navigation systems, like inaccurate heading references and degradation or loss of Doppler inputs during a flight over large water areas. Most modern DNSs combine the inherent information of Doppler measurements with the information from other navigation systems, i.e., the Inertial Reference System (IRS), the VHF (very high frequency) Omnidirectional Range and Distance Measuring Equipment (VOR/DME), or the Global Positioning System (GPS) [26]. The frequency band between 13.25 and 13.4 GHz has been internationally authorized for DNS operation. The center frequency of this band is 13.325 GHz. Two other bands, in particular, centered at 8.8 and 9.8 GHz, respectively, have been used in earlier DNS designs, while currently they are no longer applied in standalone Doppler radar design [24]. Typically, the DNS utilizes either three-beam *λ*-configured (beams 1, 2, and 3) or four-beam *x*-configured (beams 1, 2, 3, and 4) antennas, directed as shown in Figure 1 [27].

The effective beamwidth *θ_b_* of the DNS antenna is between 3° and 10° [27]. The typical mounting angle for the antenna beam axis in the vertical plane *θ*_0_ is between 15° and 30° [24]. In the horizontal plane, the mounting angle of the antenna beam axis Γ_0_ is between 15° and 45° [28]. In the inclined plane, the beam axis mounting angle *η_0_* (angle between the longitudinal axis of the antenna and the central direction of the beam) is between 65° and 80° [24]. Every DNS beam provides the same angular resolutions in the vertical and azimuthal planes, Δ*θ* and Δ*α*, respectively. A more detailed reference regarding the choice of the DNS beams parameters can be found in [27].

Operating over sea/ocean, the DNS multi-beam antenna provides the selection of the backscattered power from various directions relative to the aircraft course *ψ*, which are different from each other significantly. This key feature allows considering the DNS as a multi-beam scatterometer that we suggest here to utilize for the sea-surface wind retrieval complementary to its normal navigation functions.

### 2.2. Sea-Wind Retrieval

Blowing over the sea, winds modify properties of the microwave backscattering, so that it becomes dependent on the direction and speed of the wind. The wind speed *U* is recovered with a scatterometer, since at medium incidence angles *θ*, stronger winds results in larger NRCS $\sigma^{\circ }(U,\theta ,\alpha )$ as well as at small incidence angle, they produce smaller NRCS. As the NRCS depends on the azimuth illumination angle *α* towards the up-wind direction, the wind direction can also be measured [29]. For the scatterometer wind retrieval over the sea, the so-called “geophysical model function” is used providing an explicit relationship between the wind over water and NRCS. At medium incidence angles, the minimum requirement for the wind retrieval over water is the availability of several NRCS values (at least three NRCSs or more) corresponding to the significantly different azimuthal directions. The DNS antenna is capable of providing such NRCSs.

During the horizontal rectilinear flight, these NRCSs obtained with the roll-and-pitch-stabilized antenna beams 1, 2, 3, and 4 are $\sigma^{\circ }(U,\theta_{0},\alpha +\psi_{0.b.1})$, $\sigma^{\circ }(U,\theta_{0},\alpha +\psi_{0.b.2})$, $\sigma^{\circ }(U,\theta_{0},\alpha +\psi_{0.b.3})$, and $\sigma^{\circ }(U,\theta_{0},\alpha +\psi_{0.b.4})$, respectively, where $\psi_{0.b.1}=\Gamma_{0}$, $\psi_{0.b.2}=180^{\circ }-\Gamma_{0}$, $\psi_{0.b.3}=180^{\circ }+\Gamma_{0}$, and $\psi_{0.b.4}=360^{\circ }-\Gamma_{0}$. However, in the case of non-stabilized antenna system (or in the case of exceeding the roll or pitch maximum angle of stabilization for the stabilized antenna system), the actual incidence angles and azimuth directions of the beams are very different from their mounting angle values.

Let an aircraft use a DNS with the fixed-antenna that have four beams and perform a horizontal rectilinear flight at the altitude *H* over the mean water surface with the speed *V*. Assuming the beams to be identical and narrow enough, the current azimuthal direction of beam *N* (from beams 1, 2, 3, and 4) is *ψ_b_*_.*N*_ relative to the aircraft ground track (aircraft course), the current angle of incidence of beam *N* is *θ_b_*_.*N*_, and the NRCS corresponded to the beam *N* is $\sigma^{\circ }(U,\theta_{b.N},\alpha +\psi_{b.N})$. Let the wind blow in direction *ψ*_w_, the angle between the aircraft course *ψ* and the up-wind direction be *α*, and the geophysical model function be [29]

(1) $$\sigma^{\circ }(U,\theta ,\alpha )=A(U,\theta )+B(U,\theta )\cos\alpha +C(U,\theta )\cos(2\alpha )$$

where A(U,θ), B(U,θ) and C(U,θ) are the Fourier coefficients dependent on the speed of the wind over the sea and incidence angle, $A(U,\theta )=a_{0}(\theta )U^{\gamma_{0}(\theta )}$, $B(U,\theta )=a_{1}(\theta )U^{\gamma_{1}(\theta )}$, and $C(U,\theta )=a_{2}(\theta )U^{\gamma_{2}(\theta )}$; $a_{0}(\theta )$, $a_{1}(\theta )$, $a_{2}(\theta )$, $\gamma_{0}(\theta )$, $\gamma_{1}(\theta )$ and $\gamma_{2}(\theta )$ are the coefficients dependent on the incidence angle, radar wavelength, and polarization.

Then, the wind speed and the up-wind direction can be retrieved from the azimuth NRCSs data using the system of four equations [27]:

(2) $$\left\{ \begin{array}{ll} \sigma^{\circ }(U,\theta_{b.1},\alpha +\psi_{b.1}) & =A(U,\theta_{b.1})\\ & +B(U,\theta_{b.1})\cos(\alpha +\psi_{b.1})\\ & +C(U,\theta_{b.1})\cos(2(\alpha +\psi_{b.1})),\\ \sigma^{\circ }(U,\theta_{b.2},\alpha +\psi_{b.2}) & =A(U,\theta_{b.2})\\ & +B(U,\theta_{b.2})\cos(\alpha +\psi_{b.2})\\ & +C(U,\theta_{b.2})\cos(2(\alpha +\psi_{b.2})),\\ \sigma^{\circ }(U,\theta_{b.3},\alpha +\psi_{b.3}) & =A(U,\theta_{b.3})\\ & +B(U,\theta_{b.3})\cos(\alpha +\psi_{b.3})\\ & +C(U,\theta_{b.3})\cos(2(\alpha +\psi_{b.3})),\\ \sigma^{\circ }(U,\theta_{b.4},\alpha +\psi_{b.4}) & =A(U,\theta_{b.4})\\ & +B(U,\theta_{b.4})\cos(\alpha +\psi_{b.4})\\ & +C(U,\theta_{b.4})\cos(2(\alpha +\psi_{b.4})). \end{array}\right.$$

Further, the navigation direction of wind is retrieved as:

(3) $$\psi_{w}=\psi -\alpha \pm 180^{\circ }$$

## 3. Results and Discussion

To analyze the feasibility of the developed algorithm for the wind retrieval, a series of computer simulations using a Ku-band model (1) from [1] developed for the horizontal transmit and receive polarization was carried out. Rayleigh Power (Exponential) distribution has been used to generate the “measured” NRCSs. The mounting angles of the antenna beam axis in the horizontal plane of 45° and 15° (the highest and lowest angles from the typical range of mounting angles of the antenna beam axis in the horizontal plane) at the worst case of a cross-wind horizontal rectilinear flight with the angle of attack of −5° have been considered.

Two series of simulations have been performed. The first series of simulations focused on the incidence angle of 30° corresponding to the highest mounting angle of the antenna beam axis in the vertical plane from the typical range of DNS mounting angles. The second series of simulations focused on the incidence angle of 45°, which is higher than the typical mounting angle of the antenna beam axis in the vertical plane. The given angle of attack of −5° results in the following actual combinations of angles. At *θ*_0_ = 30°, the combinations for Γ_0_ = 45° are (*ψ_b_*_.4_ = 307° and *ψ_b_*_.1_ = 53°, *θ_b_*_.4_ = *θ_b_*_.1_ = *θ*_1_ = 27°) and (*ψ_b_*_.2_ = 142° and *ψ_b_*_.3_ = 218°, *θ_b_*_.2_ = *θ_b_*_.3_ = *θ*_2_ = 33°); for Γ_0_ = 15° are (*ψ_b_*_.4_ = 276° and *ψ_b_*_.1_ = 84°, *θ_b_*_.4_ = *θ_b_*_.1_ = *θ*_1_ = 29°) and (*ψ_b_*_.2_ = 113° and *ψ_b_*_.3_ = 247°, *θ_b_*_.2_ = *θ_b_*_.3_ = *θ*_2_ = 31°). At the same time, the combinations at *θ*_0_ = 45° for Γ_0_ = 45° are (*ψ_b_*_.4_ = 310° and *ψ_b_*_.1_ = 50°, *θ_b_*_.4_ = *θ_b_*_.1_ = *θ*_1_ = 43°) and (*ψ_b_*_.2_ = 140° and *ψ_b_*_.3_ = 220°, *θ_b_*_.2_ = *θ_b_*_.3_ = *θ*_2_ = 48°); for Γ_0_ = 15° are (*ψ_b_*_.4_ = 280° и *ψ_b_*_.1_ = 80°, *θ_b_*_.4_ = *θ_b_*_.1_ = *θ*_1_ = 44°) and (*ψ_b_*_.2_ = 110° and *ψ_b_*_.3_ = 250°, *θ_b_*_.2_ = *θ_b_*_.3_ = *θ*_2_ = 46°).

**Figure 2:**
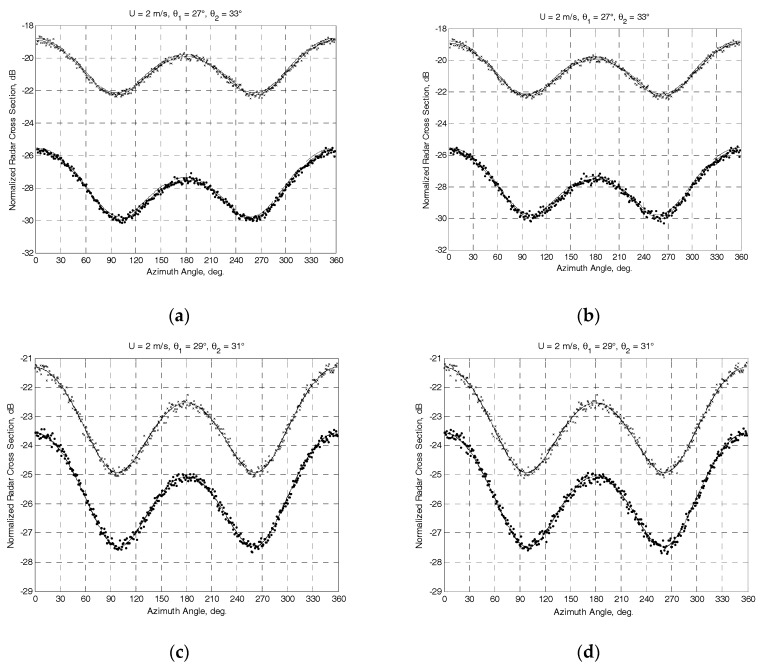
Simulation results for the mounting angle of a beam axis of 30° in the vertical plane and the angle of attack of −5° at the “true” speed of wind of 2 m/s. Solid traces show the NRCS curves according to the model (1) at the “true” wind speed. Crosses and dotted traces demonstrate the generated “measured” NRCS after integrating 1565 samples at actual incidence angles of beams *θ*_1_ and *θ*_2_, respectively. Dashed traces show the azimuth NRCS curves according to the model (1) corresponding to “measured” up-wind directions and wind speeds: (**a**) “measured” wind speed of 2 m/s and up-wind direction of 1.1° at Γ_0_ = 45° with combinations (*ψ_b_*_.4_ = 307° and *ψ_b_*_.1_ = 53°, *θ*_1_ = 27°) and (*ψ_b_*_.2_ = 142° and *ψ_b_*_.3_ = 218°, *θ*_2_ = 33°); (**b**) “measured” wind speed of 2 m/s and up-wind direction of 1.2° at the same combinations and with the instrumental noise of 0.2 dB; (**c**) “measured” wind speed of 2.01 m/s and up-wind direction of 1.5° at Γ_0_ = 15° with combinations (*ψ_b_*_.4_ = 276° and *ψ_b_*_.1_ = 84°, *θ*_1_ = 29°) and (*ψ_b_*_.2_ = 113° and *ψ_b_*_.3_ = 247°, *θ*_2_ = 31°); (**d**) “measured” wind speed of 2.01 m/s and up-wind direction of 1.7° at the same combinations and with the instrumental noise of 0.2 dB.

**Figure 3:**
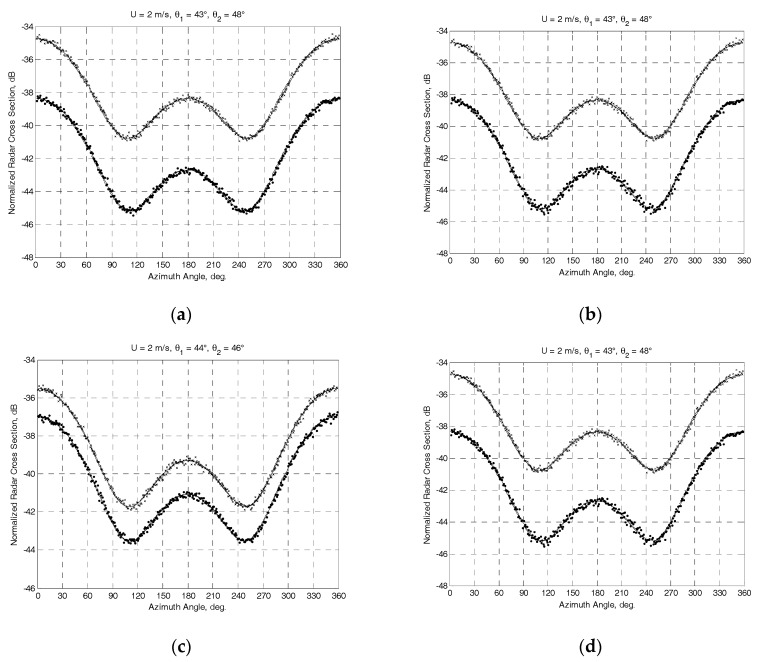
Simulation results for the mounting angle of a beam axis of 45° in the vertical plane and the angle of attack of −5° at the “true” speed of wind of 2 m/s. Solid traces show the NRCS curves according to the model (1) at the “true” wind speed. Crosses and dotted traces demonstrate the generated “measured” NRCS after integrating 1565 samples at actual incidence angles of beams *θ*_1_ and *θ*_2_, respectively. Dashed traces show the azimuth NRCS curves according to the model (1) corresponding to “measured” up-wind directions and wind speeds: (**a**) “measured” wind speed of 2 m/s and up-wind direction of 358.5° at Γ_0_ = 45° with combinations (*ψ_b_*_.4_ = 310° and *ψ_b_*_.1_ = 50°, *θ*_1_ = 43°) and (*ψ_b_*_.2_ = 140° and *ψ_b_*_.3_ = 220°, *θ*_2_ = 48°); (**b**) “measured” wind speed of 2 m/s and up-wind direction of 358.7° at the same combinations and with the instrumental noise of 0.2 dB; (**c**) “measured” wind speed of 2 m/s and up-wind direction of 359° at Γ_0_ = 15° with combinations (*ψ_b_*_.4_ = 280° и *ψ_b_*_.1_ = 80°, *θ*_1_ = 44°) and (*ψ_b_*_.2_ = 110° and *ψ_b_*_.3_ = 250°, *θ*_2_ = 46°); (**d**) “measured” wind speed of 2 m/s and up-wind direction of 358.9°at the same combinations and with the instrumental noise of 0.2 dB.

**Figure 4:**
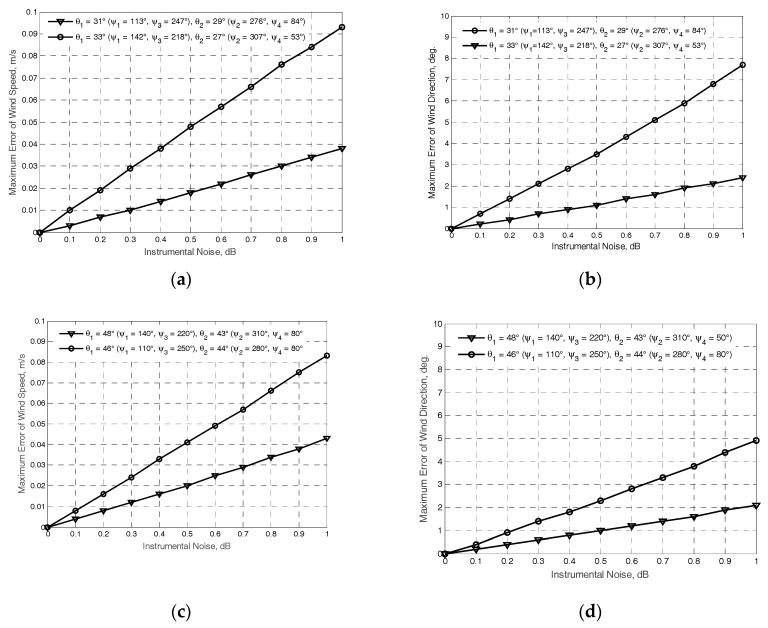
Simulation results for the maximum error dependences of the wind retrieval from the instrumental noise: (**a**) maximum errors of the wind speed at Γ_0_ = 45° with combinations (*ψ_b_*_.4_ = 307° and *ψ_b_*_.1_ = 53°, *θ*_1_ = 27°) and (*ψ_b_*_.2_ = 142° and *ψ_b_*_.3_ = 218°, *θ*_2_ = 33°) (line with rounds), and at Γ_0_ = 15° with combinations (*ψ_b_*_.4_ = 276° and *ψ_b_*_.1_ = 84°, *θ*_1_ = 29°) and (*ψ_b_*_.2_ = 113° and *ψ_b_*_.3_ = 247°, *θ*_2_ = 31°) (line with triangles); (**b**) maximum errors of the wind direction at the same combinations; (**c**) maximum error of the wind speed at Γ_0_ = 45° with combinations (*ψ_b_*_.4_ = 310° and *ψ_b_*_.1_ = 50°, *θ*_1_ = 43°) and (*ψ_b_*_.2_ = 140° and *ψ_b_*_.3_ = 220°, *θ*_2_ = 48°) (line with triangles), and at Γ_0_ = 15° with combinations (*ψ_b_*_.4_ = 280° and *ψ_b_*_.1_ = 80°, *θ*_1_ = 44°) and (*ψ_b_*_.2_ = 110° and *ψ_b_*_.3_ = 250°, *θ*_2_ = 46°) (line with triangles); (**d**) maximum errors of the wind direction at the same combinations.

The results obtained at the “true” near-surface wind speed of 2 m/s are exemplified in Figure 2 and Figure 3. The NRCS curves following model (1) at the “true” wind speed are shown by solid curves. Crosses and dotted traces demonstrate the “measured” NRCS obtained by integrating 1565 samples for each azimuthal angle with the step of one degree at the actual beam incidence angles of beams *θ*_1_ and *θ*_2_, respectively. Dashed traces show the azimuth NRCS curves accordingly to the model (1) correspondingly to the “measured” up-wind directions and wind speeds. Left columns in Figure 2 and Figure 3 represent the results without the influence of the instrumental measurement noise. As reported in [30], the typical instrumental noise of about 0.2 dB at scatterometer measurements may lead to the error of 0.5 m/s only. Right columns in Figure 2 and Figure 3 represent similar results for the 0.2 dB instrumental noise scenario. Accordingly, the influence of higher instrumental noise of up to 1 dB has also been considered in the simulations. Right panels in Figure 4 demonstrate the maximum errors of wind speed recovering as a function of the instrumental noise obtained from simulations with 100 independent instrumental noise realizations, while their left panels demonstrate the maximum errors of wind direction retrieval at the same number of independent instrumental noise realizations.

The above results clearly indicate that the DNS with a four-beam antenna that is not stabilized physically to the local horizontal is suitable for the measurement of the sea-surface wind at the typical mounting angle of the antenna beam axis in the vertical plane of 30° or higher. The accuracy of the algorithm proposed, even in the considered worst case scenario of 2 m/s wind speed and the typical mounting angle of the antenna beam axis in the horizontal plane of 15°, is within the usual accuracy range for scatterometer wind measurement. These results also indicate that DNS with the increased mounting angle of 45° for the antenna beam axis in the vertical plane provides a better usage of anisotropic properties of the sea-surface scattering at wind measurements over water.

Equipped with a combination of reliable navigation tools including multiple INSs and DNSs, modern aircraft provide very accurate roll and pitch control, especially when it comes to simple rectilinear or circular flight scenarios that are commonly performed in autopilot mode. Under calm atmospheric conditions that are optimal for measurements, both roll and pitch control accuracy is around 0.3 degrees that could be easily neglected in our calculations. When it comes to passing zones with more turbulent atmospheric conditions, short-term variability of the aircraft roll and pitch can normally be observed, before it is again stabilized by flight controls. If periods of turbulence are rather short, for practical purposes, it would be easier to eliminate these measurements from further analysis, than to develop a dedicated correction, that would appear quite complex due to nonlinear character of turbulent flows. Uncertainties in the mounting angle of the antenna beam are very low as well, typically not exceeding 15 angular minutes [24,25]. As the NRCS curves are quite smooth, especially in the horizontal plane, they have an inessential impact on wind measurement results.

Application of DNS with a non-stabilized antenna as the wind sensor over the sea surface has some limitations, which should be taken into account. The incidence angle should remain within the range of the geophysical model function validity at medium incidence angles. The incidence angle validity typically ranges from 25–30° to 55–60°. Since a non-stabilized antenna system is used, the combination of the current angle of attack, pitch, and roll should not cause the actual incidence angles of beams going out of the range of validity for the geophysical model applied.

Since at the wind retrieval, the NRCS model function (1) is used without any correction while the azimuth angular size of the selected cell is not wider than 15–20° [31], the actual resolution in the azimuthal plane should not exceed that value. At the narrowest typical DNS beamwidth of 3° in the horizontal plane, the angular resolutions in the azimuthal plane are 7.1°, 6.0°, 4.2° and 3.5° at the incidence angles of 25°, 30°, 45° and 60°, respectively. Alternatively, the widest typical DNS beamwidth in the same plane of 10° does not always provide acceptable azimuthal resolutions in the azimuthal plane. They are 23.4°, 19.9°, 14.1° and 11.6°, respectively, at the same incidence angles. Furthermore, delay selection may be further required in case of the antenna beam is not sufficiently narrow to provide the required angular resolution in the vertical plane. The presented simulation results have been obtained under the assumption of the narrow angular resolutions in both the horizontal and the vertical planes.

**Figure 5:**
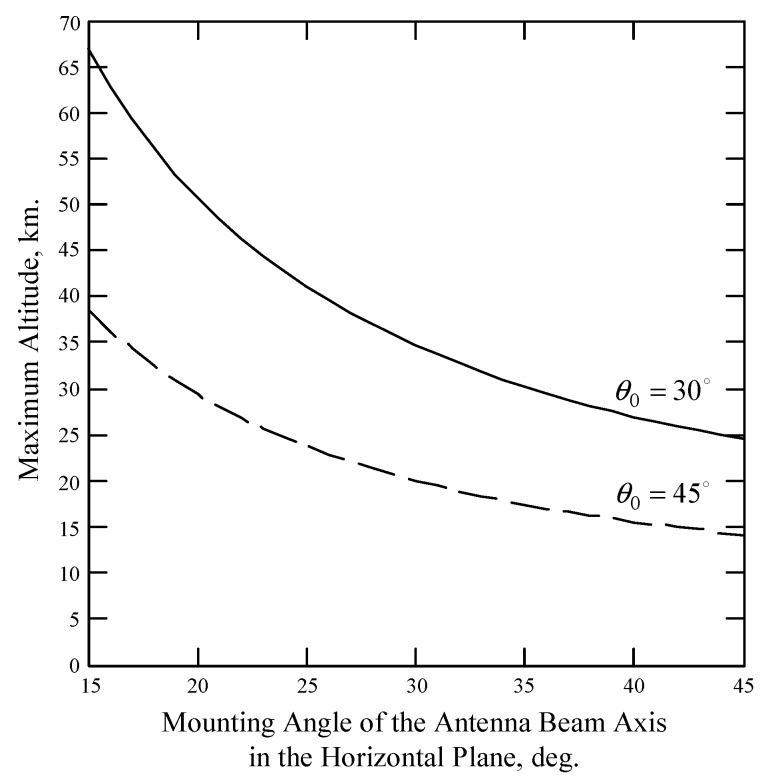
Maximum altitude of DNS wind retrieval verses mounting angle of the antenna beam axis in the horizontal plane.

The maximum altitude depends on the DNS beam geometry. Assuming the wind and wave conditions are identical in the area with a side that does not exceed 15–20 km, the maximum altitudes for the wind measurement at the mounting angle of the antenna beam axis in the vertical plane of 30° will be about 24 and 67 km at the mounting angles of the antenna beam axis in the horizontal plane of 45° and 15°, respectively (Figure 5). The maximum altitudes at the mounting angle of the antenna beam axis in the vertical plane of 45° will be lower in 1.73 times than at 30°.

## 4. Conclusions

The study has shown explicitly that the DNS equipped with the fixed-antenna (or with the track-stabilized antenna when current roll or pitch angles exceed maximum angles of stabilization) with *x*-configuration of its beams and operated as a four-beam scatterometer at a rectilinear flight can be used as a sea-wind sensor enhancing the DNS typical navigation functionality.

Since the wind scatterometer is well-calibrated, internal calibration should be implemented in the DNS for the sea-wind retrieval mode. For that, typical scatterometer internal calibration procedure can be used. The internal calibration can be achieved by coupling a small fraction of the transmitted signal into the receiving channel. The current internal calibration precision is better than 0.15 dB that results in the wind-speed error of around 0.1 m/s [32].

The horizontal transmit and receive polarization should be used, as it provides greater difference between the down-wind and up-wind NRCSs as well as between the cross-wind and up-wind NRCSs than at the vertical polarization. Increasing the mounting angle of the antenna beam axis in the vertical plane from 30° to 45° allows for the better utilization of the anisotropy of the sea surface backscattering and thus leads to providing better wind retrieval as well as allows widening the ranges of angles of attack, pitch, and roll during wind measurement by DNS. Otherwise, the highest available mounting angle of the antenna beam axis in the vertical plane from the range of its typical angles should be used. The mounting angle of the antenna beam axis in the horizontal plane also should be tending to 45° to provide NRCS sampling from significantly different directions.

The concept, algorithm and measurement principle presented here can be applied for the DNS functionality enhancement, for creating new airborne radar systems for operational measurement of winds over water as well as for ensuring the safe landing of amphibious aircraft or seaplanes on sea, especially during firefighting operations or search-and-rescue missions in the fire-risk regions or coastal areas saving people and protecting the environment.

## Abbreviations

The following abbreviations are used in this manuscript:

DNS	Doppler navigation system
GPS	global positioning system
IRS	inertial reference system
NRCS	normalized radar cross section
VHF	very high frequency
VOR/DME	VHF omnidirectional range and distance measuring equipment
